# Epidemiology of isolated preaxial polydactyly type I: Data from the Polish Registry of Congenital Malformations (PRCM)

**DOI:** 10.1186/1471-2431-13-26

**Published:** 2013-02-19

**Authors:** Anna Materna-Kiryluk, Aleksander Jamsheer, Katarzyna Wisniewska, Barbara Wieckowska, Janusz Limon, Maria Borszewska-Kornacka, Henryka Sawulicka-Oleszczuk, Ewa Szwalkiewicz-Warowicka, Anna Latos-Bielenska

**Affiliations:** 1 Chair and Department of Medical Genetics, Poznan University of Medical Sciences, Poznan, Poland; 2 Department of Preventive Medicine, Poznan University of Medical Sciences, Poznan, Poland; 3 Department of Computer Science and Statistics, Poznan University of Medical Sciences, Poznan, Poland; 4 Chair and Department of Biology and Genetics, Medical University of Gdansk, Gdansk, Poland; 5 Department of Neonatology and Neonatal Intensive Care, Medical University of Warsaw, Warsaw, Poland; 6 Department of Obstetrics and Pathology of Pregnancy, Medical University of Lublin, Lublin, Poland; 7 Regional Children’s Specialized Hospital, Olsztyn, Poland

**Keywords:** Polydactyly, Preaxial polydactyly type I, Registry

## Abstract

**Background:**

Polydactyly represents a heterogeneous group of congenital hand and foot anomalies with variable clinical features and diverse etiology. Preaxial polydactyly type I (PPD1) is the most frequent form of preaxial polydactyly. The etiology of sporadic PPD1 remains largely unknown and the relative contribution of genetic and environmental factors is not clearly defined. The primary goals of this study are twofold: (1) to examine the epidemiology and clinical features of sporadic PPD1 in comparison to a healthy control group, and (2) to contrast the characteristics of sporadic PPD1 with familial forms of isolated polydactyly.

**Methods:**

Among 2,530,349 live births registered in the Polish Registry of Congenital Malformations (PRCM), we identified 459 children with isolated sporadic PPD1 and 353 children with familial polydactyly, including 57 children with familial PPD1.

**Results:**

In comparison with the matched group of 303 controls, sporadic PPD1 cases had significantly lower birth order (P = 0.01) and birthweight (P < 0.0001). Similarly, when compared to familial cases of polydactyly, lower birth order (P = 0.047) and lower birthweight (P < 0.0001) were characteristic of sporadic PPD1 cases. Moreover, our analyses suggested several additional risk factors for sporadic PPD1, including lower paternal education levels (P = 0.01), upper respiratory tract infections during the first trimester of pregnancy (P = 0.049), and maternal history of epilepsy (P = 0.01).

**Conclusions:**

In summary, our study provides support to the hypothesis that non-genetic factors play an important role in the etiology of non-familiar PPD1.

## Background

Polydactyly represents a heterogeneous group of congenital anomalies involving hand and foot. The clinical features are highly variable and the etiology is largely unknown. Isolated polydactyly has been classified as preaxial, axial, or postaxial. While preaxial and postaxial polydactylies occur more frequently, axial forms are extremely rare. Preaxial polydactyly, defined as polydactyly on the radial side of the hands and tibial side of the feet, appears more heterogeneous than the postaxial form. There are four types of preaxial polydactyly: Type I (PPD1): thumb/hallux polydactyly, type II (PPD2): polydactyly of triphalangeal thumb, type III (PPD3): polydactyly of index finger, and type IV (PPD4): polysyndactyly. Among the four types, PPD1 is the most frequent form. Its characteristic feature is doubling of one or more skeletal components of a thumb or hallux. In its mildest form, it can manifest as a widening or small bifurcation of a thumb or hallux.

PPD1 exhibits differences in occurrence across various ethnic groups, but presently its genetics are not well understood. A small number of familial cases have been linked to the disruption of the Shh signal transduction pathway, which plays a major role in antero-posterior patterning of limbs [1]. Nevertheless, based on the previous epidemiologic investigations, environmental factors cannot be disregarded in the determination of this phenotype. For example, sporadic PPD1 has been observed more frequently in infants of diabetic mothers [2] and in babies with thalidomide embryopathy [3].

We undertake this work to better delineate the environmental factors associated with higher risk of sporadic PPD1. The goals of this work are twofold: (1) to examine the clinical features and the epidemiology of sporadic PPD1 in Poland, and (2) to compare epidemiologic characteristics of sporadic PPD1 with the familial forms of isolated polydactyly (more likely attributable to genetic causes).

## Methods

### Cases

Among 2,530,349 live births registered in the Polish Registry of Congenital Malformations (PRCM), we identified 459 children with isolated sporadic PPD1 and 353 children with familial polydactyly (including 57 children with familial PPD1). The data was collected between years 1998 and 2007 from 13 geographic regions of Poland, comprising approximately 85% of the total area of the country. The following regions were analyzed: dolnoslaskie, kujawsko-pomorskie, lubuskie, lodzkie, opolskie, pomorskie, warminsko-mazurskie, wielkopolskie, zachodniopomorskie (between 1998 and 2007), slaskie (between 2001 and 2007), lubelskie, podkarpackie (between 2002 and 2007) and mazowieckie (between 2004 and 2007). Standardized congenital malformation reporting questionnaires were used as a primary information source. The questionnaires were completed by physicians from neonatal and pediatric wards, and the information was entered into the Registry database. The organization of PRCM has been described in detail elsewhere [4]. The data analyzed in this study was collected as part of the PRCM registry, a member of the Eurocat network of population-based registries for the epidemiological surveillance of congenital anomalies. The authors include the founders and the coordinators of the PRCM registry. The authors have designed the data collection system, collected the primary data, managed the database, and performed all analyses presented in this study. These data will also be made available by the authors to any interested investigators upon a written request and approval of the local ethics committee.

The main criterion for the diagnosis was the presence of isolated sporadic defects: polydactyly type I (thumb/hallux polydactyly). Exclusion criteria included: preaxial polydactyly type II, III, IV, familial preaxial polydactyly type I, postaxial polydactyly, and complex polydactyly (simultaneous pre- and postaxial). All known syndromes and chromosomal aberrations associated with multiple birth defects and polydactyly were also excluded.

### Controls

Since healthy individuals without any malformation are not routinely reported to the registry, the control group was selected among individuals with a benign isolated mild ankyloglossia. Mild ankyloglossia (short lingular frenulum without clinical consequences), or “tongue tied”, is the most common and benign oral anomaly caused by an unusually short thickened lingual frenulum, and thus it is highly unlikely to be etiologically related to polydactyly or limb malformations. To be eligible for inclusion in the control group, individuals with mild ankyloglosia, had no other congenital malformations, and were reported to the registry at the same time and from the same general area of residence as the polydactyly cases. Moreover, in order to assure that our controls were representative, they were matched to the general Polish newborn population with respect to sex distribution, maternal age, and maternal education level. The matching was performed using a random sampling procedure with a proportionate allocation within each stratum (a sampling fraction within each stratum was proportional to that of the general population). The stratified sampling was implemented in Excel (Microsoft, 2007). The data on the characteristics of the general population were obtained from the Central Polish Statistical Office [5]. In total, 303 of 612 initially available individuals with isolated mild ankyloglosia met our matching criteria and were included as controls in this study.

### Statistical analysis

The univariate (unadjusted) comparisons of demographic and clinical risk factors across different study groups were conducted using the Fisher’s exact test (categorical variables) or Student’s t-test (continuous variables). Adjusted analyses were performed within the logistic regression framework, using StatXact and LogXact software version 8.0.0 (Cytel Studio). P-values <0.05 were considered statistically significant.

## Results

Based on the PRCM reports, we estimated the incidence of sporadic PPD1 in Poland at 181.4 cases per million live birth-years. The sporadic cases of PPD1 were predominantly unilateral (92.8%) and had a strong predilection for the hands (94.8%). Among unilateral cases, sporadic thumb polydactyly occurred more frequently on the right side (61.2%, P < 0.0001). We also noted a trend for sporadic hallux polydactyly to occur more commonly on the left side (71.4%, P = 0.08). These observations are summarized in Table 1.

**Table 1:** Laterality of limb defects in sporadic preaxial polydactyly type I (PPD1), Polish Registry of Congenital Malformations, 1998-2007

**Characteristic**	**Sporadic PPD1 N = 459**			
	**Thumb**		**Hallux**	
	**N = 435**	**P-value**	**N = 24**	**P-value**
Unilateral	405		21	
Left	157 (38.8%)	<0.0001	15 (71.4%)	0.08 (NS)
Right	248 (61.2%)		6 (28.6%)	
Bilateral	18		3	
Unknown	12		0	

Statistical significance of exact test for proportion R:L ratio = 1.

NS: Not significant.

The incidence of familial polydactyly in Poland was estimated at 139.5 cases per million live birth-years. In total, approximately 50% of index cases with positive family history had at least one affected parent, 26% had at least one affected sibling, and 17% had at least one affected grandparent. The exact numbers of affected first- and second-degree relatives by type of polydactyly is summarized in Table 2.

**Table 2:** The number of affected first- and second-degree relatives of index cases with familial polydactyly reported to the Polish Registry of Congenital Malformations, 1998-2007

**Type of Familial Polydactyly**	**Relatives I**^**o**^		**Relatives II**^**o**^	
	**Siblings**	**Parents**	**Grandparents**	**Parents' siblings**
Preaxial - PPD (N = 78)	15 (19.2%)	31 (39.7%)	16 (20.5%)	17 (21.8%)
PPD1 (N = 57)	14	17	10	12
PPD2 (N = 1)	0	1	1	0
PPD3 (N = 5)	0	4	0	0
PPD4 (N = 14)	1	9	4	5
PPD1 + PPD2 (N = 1)	1	0	1	0
Postaxial (N = 148)	38 (25.7%)	77 (52.0%)	24 (16.2%)	40 (27.0%)
Unknown (N = 127)	39 (30.7%)	70 (55.1%)	21 (16.5%)	29 (22.8%)
Total (N = 353)	92 (26.1%)	178 (50.4%)	61 (17.3%)	86 (24.3%)

The characteristics of cases with sporadic PPD1, familial cases of polydactyly, and the controls are summarized and compared in Table 3. Interestingly, both types of polydactyly were more common in males (male-to-female ratio of 1.29). Low birthweight was the most significant risk factor associated with sporadic PPD1. The risk for sporadic PPD1 decreased by approximately 31% with each extra 500 g gain in body weight (OR = 0.69, [95% CI: 0.61-0.79 per 500 g body weight], P < 0.0001). Other associated risk factors for sporadic PPD1 included earlier pregnancy and lower birth order (OR = 0.87 per each subsequent pregnancy, [95% CI: 0.77-0.97], P = 0.02 and OR = 0.84 per each subsequent birth, [95% CI: 0.74-0.96], P = 0.01). We also observed a trend for a higher risk of sporadic PPD1 with lower paternal education (OR = 0.81 per each education level, [95% CI: 0.64-1.01], P = 0.06). None of the above trends or associations was observed for the familial forms of polydactyly.

**Table 3:** Demographic and clinical characteristics of sporadic preaxial polydactyly type I (PPD1), familial polydactyly (all types), and healthy control group

**Characteristic**	**Controls**	**Sporadic PPD1**		**Familial Polydactyly**	
	**N = 303**^**$**^	**N = 459**^**$**^		**N = 353**^**$**^	
	**Count (%)**	**Count (%)**	**P-value OR [95% CI]**	**Count (%)**	**P-value OR [95% CI]**
*Gender*					
Female (reference)	135 (44.6%)	200 (43.6%)	0.83 (NS)	154 (43.6%)	0.84 (NS)
Male	168 (55.4%)	257 (56%)	1.03 [0.77, 1.38]	198 (56.1%)	1.03 [0.76, 1.41]
*Residence type*					
Urban (reference)	179 (60.3%)	282 (61.8%)	0.67 (NS)	210 (60%)	0.94 (NS)
Rural	118 (39.7%)	174 (38.2%)	0.94 [0.69, 1.26]	140 (40%)	1.01 [0.74, 1.39]
*Maternal age*^*#*^					
19 years and less	20 (6.6%)	36 (7.8%)		28 (7.9%)	
20-24	81 (26.7%)	147 (32%)		94 (26.6%)	
25-29	110 (36.3%)	147 (32%)	0.57 (NS)	128 (36.3%)	0.95 (NS)
30-34	63 (20.8%)	91 (19.8%)	0.99 [0.95, 1.03]	61 (17.3%)	1.00 [0.96, 1.04]
35-39	25 (8.3%)	28 (6.1%)		28 (7.9%)	
40 years and more	4 (1.3%)	5 (1.1%)		13 (3.7%)	
*Paternal age*^*#*^					
19 years and less	7 (2.3%)	11 (2.4%)		6 (1.7%)	
20-24	36 (11.9%)	81 (17.6%)		52 (14.7%)	
25-29	98 (32.3%)	154 (33.6%)		115 (32.6%)	
30-34	74 (24.4%)	115 (25.1%)	0.27 (NS)	84 (23.8%)	0.95 (NS)
35-39	39 (12.9%)	54 (11.8%)	0.98 [0.95, 1.02]	49 (13.9%)	1.00 [0.97, 1.04]
40-44	20 (6.6%)	24 (5.2%)		26 (7.4%)	
45-49	5 (1.7%)	5 (1.1%)		9 (2.5%)	
50 years and more	2 (0.7%)	0 (0%)		3 (0.8%)	
Maternal education^*^					
Elementary or incomplete elementary	43 (14.2%)	63 (13.7%)		41 (11.6%)	
Vocational	91 (30%)	115 (25.1%)	0.14 (NS)	105 (29.7%)	0.67 (NS)
Secondary	108 (35.6%)	160 (34.9%)	1.16 [0.95, 1.42]	106 (30%)	1.05 [0.85, 1.30]
Higher	61 (20.1%)	94 (20.5%)		87 (24.6%)	
Paternal education^*^					
Elementary or incomplete elementary	21 (6.9%)	51 (11.1%)		32 (9.1%)	
Vocational	128 (42.2%)	196 (42.7%)	0.06 (NS)	135 (38.2%)	0.50 (NS)
Secondary	83 (27.4%)	119 (25.9%)	0.81 [0.64, 1.01]	96 (27.2%)	1.09 [0.86, 1.38]
Higher	36 (11.9%)	55 (12%)		65 (18.4%)	
*Birthweight* [per 500 g body weight]					
500-999 g	1 (0%)	2 (0.4%)		1 (0.3%)	
1000-1499	1 (0.3%)	6 (1.3%)		1 (0.3%)	
1500-1999	9 (0.3%)	9 (2%)		4 (1.1%)	
2000-2499	46 (3%)	25 (5.4%)	<0.0001	10 (2.8%)	0.32 (NS)
2500-2999	101 (15.2%)	100 (21.8%)	0.69 [0.61, 0.79]	46 (13%)	0.93 [0.81, 1.07]
3000-3499	102 (33.3%)	175 (38.1%)		140 (39.7%)	
3500-3999	34 (33.7%)	101 (22%)		102 (28.9%)	
4000-4499	7 (11.2%)	29 (6.3%)		41 (11.6%)	
4500 g or more	0 (2.3%)	4 (0.9%)		4 (1.1%)	
*Pregnancy length*					
under 28 weeks	0 (0%)	1 (0.2%)		1 (0.3%)	
28-31	1 (0.3%)	7 (1.5%)	0.57 (NS)	3 (0.8%)	0.48 (NS)
32-36	15 (5%)	26 (5.7%)	0.98 [0.90, 1.06]	15 (4.2%)	1.03 [0.95, 1.13]
37-41	271 (89.4%)	397 (86.5%)		305 (86.4%)	
42 weeks or more	8 (2.6%)	20 (4.4%)		18 (5.1%)	
*Pregnancy order*					
1	126 (41.6%)	217 (47.3%)		155 (43.9%)	
2	87 (28.7%)	147 (32%)		115 (32.6%)	
3	45 (14.9%)	48 (10.5%)	0.02	39 (11%)	0.42 (NS)
4	28 (9.2%)	29 (6.3%)	0.87 [0.77, 0.97]	15 (4.2%)	0.95 [0.85, 1.07]
5	13 (4.3%)	5 (1.1%)		12 (3.4%)	
6	2 (0.7%)	6 (1.3%)		5 (1.4%)	
7 or more	2 (0.7%)	4 (0.9%)		7 (2%)	
*Birth order*					
1	135 (44.6%)	237 (51.6%)		166 (47%)	
2	97 (32%)	152 (33.1%)		117 (33.1%)	
3	36 (11.9%)	37 (8.1%)	0.01	35 (9.9%)	0.62 (NS)
4	29 (9.6%)	19 (4.1%)	0.84 [0.74, 0.96]	12 (3.4%)	0.97 [0.86, 1.10]
5	3 (1%)	5 (1.1%)		8 (2.3%)	
6	1 (0.3%)	4 (0.9%)		6 (1.7%)	
7 or more	2 (0.7%)	2 (0.4%)		4 (1.1%)	
*Number of spontaneous abortions for 2nd or further pregnancy*					
No (reference)	135 (76.3%)	182 (75.2%)	0.98 (NS)	155 (78.3%)	0.35 (NS)
Yes	42 (23.7%)	57 (23.6%)	0.99 [0.63, 1.57]	38 (19.2%)	0.79 [0.48, 1.30]

^$^ Marginal totals for some variables may be different because of missing values.

NS: Not significant.

^#^ Common model of logistic regression of parents’ age.

^*^ Common model of logistic regression of parent’s education.

Next, we directly compared the sporadic and familial forms of polydactyly (Table 4). Here, we again noted higher risk of sporadic PPD1 with low birthweight (OR = 0.74, [95% CI: 0.65-0.84 per 500 g body weight], P < 0.0001), lower birth order (OR = 0.89, [95% CI: 0.78-0.998], P = 0.047), and lower paternal education levels (OR = 0.76, [95% CI: 0.62-0.93], P = 0.01). The sex distribution, urban/rural residence, maternal education and number of spontaneous abortions were not different between the two case groups.

**Table 4:** Demographic and clinical characteristics of sporadic preaxial polydactyly type I (PPD1) and familial polydactyly (all types)

**Characteristic**	**Sporadic PPD1**	**Familial Polydactyly**	
	**N = 459**^**$**^	**N = 353**^**$**^	**P-value**
			**OR [95% CI]**
	**Count (%)**	**Count (%)**	
*Gender*			
Female (reference)	200 (43.6%)	154 (43.6%)	0.99 (NS)
Male	257 (56%)	198 (56.1%)	1.00 [0.75, 1.32]
*Residence type*			
Urban (reference)	282 (61.8%)	210 (60%)	0.60 (NS)
Rural	174 (38.2%)	140 (40%)	0.93 [0.70, 1.23]
*Maternal age*^*#*^			
19 years and less	36 (7.8%)	28 (7.9%)	
20-24	147 (32%)	94 (26.6%)	
25-29	147 (32%)	128 (36.3%)	0.55 (NS)
30-34	91 (19.8%)	61 (17.3%)	0.99 [0.95, 1.03]
35-39	28 (6.1%)	28 (7.9%)	
40 years and more	5 (1.1%)	13 (3.7%)	
*Paternal age*^*#*^			
19 years and less	11 (2.4%)	6 (1.7%)	
20-24	81 (17.6%)	52 (14.7%)	
25-29	154 (33.6%)	115 (32.6%)	
30-34	115 (25.1%)	84 (23.8%)	0.23 (NS)
35-39	54 (11.8%)	49 (13.9%)	0.98 [0.95, 1.01]
40-44	24 (5.2%)	26 (7.4%)	
45-49	5 (1.1%)	9 (2.5%)	
50 years and more	0 (0%)	3 (0.8%)	
Maternal education^*^			
Elementary and incomplete elementary	63 (13.7%)	41 (11.6%)	
Vocational	115 (25.1%)	105 (29.7%)	0.32 (NS)
Secondary	160 (34.9%)	106 (30%)	1.10 [0.91, 1.33]
Higher	94 (20.5%)	87 (24.6%)	
Paternal education^*^			
Elementary and incomplete elementary	51 (11.1%)	32 (9.1%)	0.01
Vocational	196 (42.7%)	135 (38.2%)	0.76 [0.62, 0.93]
Secondary	119 (25.9%)	96 (27.2%)	
Higher	55 (12%)	65 (18.4%)	
*Birthweight* [per 500 g body weight]			
500-999 g	2 (0.4%)	1 (0.3%)	
1000-1499	6 (1.3%)	1 (0.3%)	
1500-1999	9 (2%)	4 (1.1%)	
2000-2499	25 (5.4%)	10 (2.8%)	<0.0001
2500-2999	100 (21.8%)	46 (13%)	0.74 [0.65, 0.84]
3000-3499	175 (38.1%)	140 (39.7%)	
3500-3999	101 (22%)	102 (28.9%)	
4000-4499	29 (6.3%)	41 (11.6%)	
4500 g and more	4 (0.9%)	4 (1.1%)	
*Pregnancy length*			
under 28 weeks	1 (0.2%)	1 (0.3%)	
28-31	7 (1.5%)	3 (0.8%)	0.21 (NS)
32-36	26 (5.7%)	15 (4.2%)	0.95 [0.89, 1.03]
37-41	397 (86.5%)	305 (86.4%)	
42 weeks and more	20 (4.4%)	18 (5.1%)	
*Pregnancy order*			
1	217 (47.3%)	155 (43.9%)	
2	147 (32%)	115 (32.6%)	
3	48 (10.5%)	39 (11%)	0.13 (NS)
4	29 (6.3%)	15 (4.2%)	0.92 [0.83, 1.03]
5	5 (1.1%)	12 (3.4%)	
6	6 (1.3%)	5 (1.4%)	
7 and further	4 (0.9%)	7 (2%)	
*Birth order*			
1	237 (51.6%)	166 (47%)	
2	152 (33.1%)	117 (33.1%)	
3	37 (8.1%)	35 (9.9%)	0.047
4	19 (4.1%)	12 (3.4%)	0.89 [0.78, 0.998]
5	5 (1.1%)	8 (2.3%)	
6	4 (0.9%)	6 (1.7%)	
7 and further	2 (0.4%)	4 (1.1%)	
*Number of spontaneous abortions for 2nd or further pregnancy*			
No (reference)	182 (75.2%)	155 (78.3%)	0.30 (NS)
Yes	57 (23.6%)	38 (19.2%)	1.28 [0.80, 2.04]

^$^ Marginal totals for some variables may be different because of missing values.

NS: Not significant.

^#^ Common model of logistic regression of parents’ age.

^*^ Common model of logistic regression of parent’s education.

Lastly, we performed the analysis of pregnancy complications and chronic maternal conditions (Table 5). Overall, the reported complications were rare across the three groups, thus our analysis may be underpowered. However, several interesting patterns arouse from this analysis. First, the pregnancy complications of oligohydramnios and polyhydramnios were absent in the healthy control group. However, oligohydramnios was more common in both polydactyly groups, with frequencies comparable between the sporadic and familial forms (1.5% and 1.7%, respectively). In contrast, polyhydramnios appeared exclusively in the familial cases (1.1%). Additionally, our analyses suggested that upper respiratory tract infections during the first trimester of pregnancy were associated with an increased risk of sporadic PPD1 (4.1% vs. 1.7%, P = 0.049), but not the familial forms of polydactyly (P = 0.11). We also noted a more frequent occurrence of epilepsy in mothers of children with PPD1 (1.7%) when compared to the familial forms (0%) or healthy controls (0.3%). Importantly, maternal smoking history during the first trimester of pregnancy was not appreciably associated with the risk of either form of polydactyly.

**Table 5:** Differences in pregnancy complications between the controls, sporadic PPD1, and familial polydactyly (all types)

**Characteristics**	**Controls**	**Sporadic PPD1**	**Familial Polydactyly**	**Sporadic vs. Controls**	**Familial vs. Controls**	**Sporadic vs. Familial**
	**N = 303**^**$**^	**N = 459**^**$**^	**N = 353**^**$**^			
	**Count (%)**	**Count (%)**	**Count (%)**	**P-value**	**P-value**	**P-value**
				**(Fisher's Exact Test)**	**(Fisher's Exact Test)**	**(Fisher's Exact Test)**
Pregnancy pathology						
Oligohydramnios	0 (0%)	7 (1.5%)	6 (1.7%)	0.03	0.02	0.84 (NS)
Polyhydramnios	0 (0%)	0 (0%)	4 (1.1%)	1.00 (NS)	0.09 (NS)	0.035
Gestosis	13 (4.3%)	15 (3.3%)	9 (2.5%)	0.45 (NS)	0.22 (NS)	0.56 (NS)
Upper respiratory tract infections during 1st trimester of pregnancy	5 (1.7%)	19 (4.1%)	13 (3.7%)	0.049	0.11 (NS)	0.76 (NS)
Urinary system infections during 1st trimester of pregnancy	3 (1%)	5 (1.1%)	3 (0.8%)	0.90 (NS)	0.88 (NS)	0.77 (NS)
Mother’s chronic diseases						
Hypothyroidism	0 (0%)	4 (0.9%)	2 (0.6%)	0.13 (NS)	0.30 (NS)	0.64 (NS)
Epilepsy	1 (0.3%)	8 (1.7%)	0 (0%)	0.08 (NS)	0.46 (NS)	0.01
Smoking during 1st trimester of pregnancy	22 (7.3%)	39 (8.5%)	25 (7.1%)	0.91 (NS)	0.33 (NS)	0.32 (NS)
Exposure to ionizing radiation during 1st trimester of pregnancy	1 (0.3%)	2 (0.4%)	1 (0.3%)	0.99 (NS)	0.80 (NS)	0.75 (NS)

^$^ Marginal totals for some variables may be different because of missing values.

NS: Not significant.

## Discussion

Recent advances in human genetics led to successful discovery of molecular etiology underlying several types of familial polydactyly [1,6-9]. However, the etiology of sporadic PPD1 remains largely unexplained and the relative contribution of genetic and environmental factors is not clear. Our results suggest that environmental factors may play an important role in the determination of this phenotype.

The PRCM collects data on congenital malformations diagnosed among Polish children less than 2 years of age. The large numbers of polydactyly cases reported to PRCM enabled us to perform a well-powered comparison between familial and sporadic forms of this defect. In addition, we included a well-characterized group of healthy control births to further validate risk factors specific to sporadic PPD1. Nevertheless, several limitations of our study need to be mentioned. Although the PRCM currently monitors both live births and stillbirths, and multiple-source surveillance systems ensure the completeness of data on congenital malformations in live births, the data on the congenital malformations in stillbirths is incomplete. Because of this limitation, only live birth cases of isolated congenital malformations were used in our analysis. Other limitations stem from the intrinsic properties of registry data that may be susceptible to under-reporting or misdiagnosis. For example, there is a possibility that some forms of polydactyly, such as PPD4 with incomplete expression in lower extremities or without syndactyly, could have been misclassified as PPD1. Another limitation pertains to the nature of our control group. Although carefully matched to the general Polish population based on the demographic characteristics, the controls carried a diagnosis of a benign defect of lingual frenulum. There is no known association of this defect with polydactyly, or with any of the maternal or fetal characteristics under investigation in our study, thus it is highly unlikely that this limitation confounded our results. Finally, our study is based on reports from a single country with predominantly white/Caucasian population, thus our data may not be generalizable to other nationalities or ethnicities.

Despite these limitations, our data demonstrate that lower birthweight is a very strong and highly significant risk factor only for sporadic PPD1, but not for the familial forms of polydactyly. Low birthweight is correlated with many environmental and socio-economic factors, such as unemployment, lower occupational status, or single mother status [10,11]. Therefore, our findings strongly argue for a role of environmental influence on the etiology of PPD1. Notably, the association of low birthweight with increased risk of isolated PPD1 has been reported previously [12].

Our data also suggests that lower level of the paternal education increases the risk of PPD1. The level of education represents another marker of socio-economic status, and is inversely correlated with certain health behaviors, such as smoking or drug use [13,14]. This factor has not been previously examined in relationship to the risk of polydactyly, thus additional studies are needed to validate our observations.

The effect of pregnancy complications and chronic medical conditions in mothers of children with polydactyly has been studied previously. One of the largest studies of 5,171 children with a variety of digital anomalies including polydactyly, syndactyly and adactyly demonstrated that the mothers of these children were more likely to suffer from anemia, cardiac disease, lung disease, oligohydramnios, polyhydramnios, or pregnancy-associated hypertension [15]. Other implicated maternal risk factors include vaginal bleeding in the first trimester [12], and diabetes [2,15,16]. In this study, there were no PPD1 cases born to mothers with diabetes type I or II, thus it is unlikely to be an important risk factor. However, among mothers of children with sporadic PPD1 we observed a trend for increased frequency of epilepsy and upper respiratory infections during the 1^st^ trimester of pregnancy. Finally, we confirmed that oligohydramnios occurred more frequently in both sporadic and familial forms of polydactyly compared to healthy control births. Although oligohydramnios classically results from congenital abnormalities of the kidney or urinary tract, and can lead to well-defined deformation sequences [17,18], it has been described in association with other congenital malformations, such as digestive tract and cardiac malformations [19]. The etiologial basis for the observed association of oligohydramios with polydactylies remains unexplained.

A large body of scientific literature implicates genetic factors in the etiology of preaxial polydactyly [1,6-9]. Compared to other types of polydactyly, PPD1 has a relatively low familial recurrence [20,21]. In our study, 11% of all PPD1 cases were familial. However, family aggregation studies report that different types of preaxial polydactyly frequently occur within the same family. For example, extra hand digit, triphalangeal thumb, or duplication of the big toe may segregate within the same family, thus might represent manifestations of the same autosomal dominant mutation. This pleiotropic effect of causal mutations may be explained by the presence of additional environmental modifiers that may alter phenotypic expression of such genetic defects [22,23]. Importantly, our study suggests several environmental factors that may contribute to the expression of digital defects, including low birthweight, lower paternal education status, and selected pregnancy complications, such as oligohydramios.

## Conclusions

In summary, our study provides additional support for the hypothesis that non-genetic factors play an important role in the etiology of sporadic PPD1. Low birthweight is one of the most strongly associated risk factors for sporadic PPD1 in our study. This observation, however, will require validation in independent case–control cohorts.

## Competing interests

The authors declare no competing interests.

## Authors’ contributions

AM-K: Study conception, design, and implementation. AJ, KW: Literature review and manuscript preparation. BW: Statistical analysis. JL: Data collection from Pomorskie administrative region. MB-K: Data collection from Mazowieckie administrative region. HS-O: Data collection from Lubelskie administrative region. ES-W: Data collection from Warmińsko-mazurskie administrative region. AL-B: Supervision of the field activities. All authors read and approved the final manuscript.

## Pre-publication history

The pre-publication history for this paper can be accessed here:

http://www.biomedcentral.com/1471-2431/13/26/prepub
